# The Burden of Undiagnosed Diabetes Mellitus in Adult African Population: A Systematic Review and Meta-Analysis

**DOI:** 10.1155/2019/4134937

**Published:** 2019-04-28

**Authors:** Daniel Asmelash, Yemane Asmelash

**Affiliations:** ^1^Department of Clinical Chemistry, School of Biomedical and Laboratory Sciences, College of Medicine and Health Sciences, University of Gondar, Gondar, Ethiopia; ^2^Department of Statistics, College of Computational and Natural Science, Aksum University, Aksum, Ethiopia

## Abstract

**Background:**

The prevalence of diabetes is rapidly increasing in Africa. Type two diabetes may remain undetected for many years, leading to severe complications and healthcare costs. This underlines the importance of understanding the magnitude of undiagnosed diabetes in different populations of Africa. This study is intended to summarize and pool the results of community-based studies to provide a continental level estimate of the undiagnosed diabetes mellitus.

**Methods:**

We searched MEDLINE/PubMed, HINARI, Cochrane Library, and Google Scholar for community-based studies on diabetes mellitus in Africa. Descriptive information for the original studies was presented in a table, and the quantitative results were presented in forest plots. The Cochran's *Q* test and *I*^2^ test statistic were used to test heterogeneity across studies. The pooled prevalence of undiagnosed diabetes and subgroup analyses within urban and rural population and diagnostic methods were computed by a random effects model from 2011 to 2017.

**Results:**

One hundred fifty-seven articles were identified through electronic searching using keywords. Of these, seventeen studies, with a total population of 20,350, met the inclusion criteria. A random effects meta-analysis showed that the pooled prevalence of undiagnosed diabetes mellitus in African population was 5.37% (95% CI: 4.57, 6.81). The pooled prevalence from subgroup analyses indicated that undiagnosed diabetes mellitus in the urban population (8.68%, 95% CI: 5.33, 12.03) is twice higher than that in the rural population (3.93%, 95% CI: 2.91, 4.95). The prevalence of UDM by OGTT (8.84%, 95% CI: 1.95, 15.73) was higher than that by the FPG diagnostic method (4.54%, 95% CI: 3.59, 5.49).

**Conclusion:**

This study found high proportions of undiagnosed diabetes mellitus in different communities of the African countries. Policy makers must consider diagnostic strategies to improve screening for the undiagnosed diabetes mellitus cases for effective care, which can bring about a substantial reduction in diabetes-related complications and mortality. This review is registered with PROSPERO registration number CRD42018092637.

## 1. Introduction

Diabetes mellitus (DM) with other noncommunicable diseases is responsible for an increasing burden of diseases in developing countries. In Sub-Saharan Africa, noncommunicable diseases are predicted to exceed infectious diseases by the year 2030 [1, 2].

The International Diabetes Federation estimates that there are approximately 425 million adults (20-79 years) who were living with diabetes in 2017 with a projected increase of 629 million by 2045 [3]. Globally, 45.8% of all diabetes cases, or 174.8 million people, are estimated to have undiagnosed diabetes mellitus (UDM) in 2013 [4]. In 2013, diabetes was responsible for 74.9 thousand deaths, which is the seventh leading cause of death, and 1.85 million years living with disability, which is the eighth leading cause of disability [5].

In addition to a health burden, diabetes-related health expenditures incur heavy cost on individuals, health systems, and governments. The global health expenditure on diabetes is expected to total at least 376 billion USD in 2010 and 490 billion USD in 2030. Globally, 12% of the health expenditures are anticipated to be spent on diabetes in 2010. UDM causes an additional cost of 2864 USD which was spent per person per year, and this is due to higher diabetic complication among UDM cases [6].

There are factors for DM cases that remain undiagnosed for many years, which include poor health systems, lack of awareness in the general population and health professionals, and slow onset of the symptoms or progression of type 2 diabetes [4, 7]. UDM is characterized by uncontrolled elevated blood glucose, which leads to the development of micro- and macrovascular complications, including neuropathy, nephropathy, retinopathy, coronary artery disease, stroke, and peripheral vascular disease [8]. The finding from a study done in the USA showed that up to 41.7% of adults with newly diagnosed diabetes have developed chronic kidney disease [9].

Studies around the world reported a different level in the prevalence of UDM. The prevalence of UDM in studies done on the general population was 7% in India [10], 5.9% in Qatar [11], 4.1% in the USA (28.6% of all diabetes cases) [5], 5.1% in Iran (56% of all diabetes cases) [12], 2.9% in Russia (53% of all diabetes cases) [13], and 4.1% in China [14].

In Africa, the prevalence of UDM is not consistent in the different countries as a result of a difference in social, economic, and genetic disparities. The prevalence of UDM in North Africa ranged from 18% to 75% of all diabetes cases [15]. Moreover, the prevalence of UDM in different regions of Africa were shown as follows: 9% in Tanzania [16], 7.2%, 11.5%, 5%, 2.3%, 3.8%, and 2.13% in Ethiopia [1, 17–21], and 2.6% and 5.97% in North Sudan [22] (East African studies); 3.19% in Guinea [23], 6.3% in Cameroon [24], 4.77% in Mauritania [25], 4.64% in Senegal [26], and 7% and 4.6% in Nigeria [27, 28]) (West African studies); 18.1% in South Africa [29]; and 4.2% in Egypt (North Africa) [30].

The early detection and intervention of DM have an enormous benefit, which is only possible when there is evidence showing the magnitude and risks of diabetes [1]. While the existence of UDM has long been recognized, wide-reaching awareness among the general public, physicians, and policy makers is lacking and there are limited reliable and comparable data available. Given the fact that UDM has been rising in African countries, this meta-analysis is designed to summarize the most currently available evidence among adult African populations.

## 2. Methods

### 2.1. Literature Search Strategy/Data Source

A systematic meta-analysis was done using published articles on the prevalence of UDM in Africa. The studies were found through Internet searches using the PubMed, Google Scholar, HINARI, and Cochrane Library databases. The exploration was done using the following keywords individually or in combination: Undiagnosed or (Newly diagnosed)) AND (Diabetes mellitus or (DM)) AND (Prevalence or (Burden)) AND (Africa). Only articles written in English were considered. The searching of articles was carried out from December 2017 to February 2018, and research articles done in the last 10 years from 2008 to 2018 were included in the meta-analysis to determine the magnitude of UDM among adult populations in Africa.

### 2.2. Study Selection

Studies were selected for the meta-analysis if they were community-based studies done in Africa and stated the prevalence of UDM. After finding all the articles from Internet searches, all papers were then assessed for eligibility by two independent researchers based on the inclusion criteria. Difference between the two researchers was resolved through discussion and consensus. Lastly, studies that met all of the following criteria were included in the meta-analysis: cross-sectional studies that were conducted in the age group of 15 years and above and done in Africa, studies that used fasting blood glucose (FPG), hemoglobin A1c (HbA1c), and oral glucose tolerance test (OGTT) for classification of diabetes mellitus, studies that reported the prevalence of UDM, and studies that used original data and a random sampling technique.

### 2.3. Data Extraction Tool

Data extraction was done using a standardized and pretested format. Data extraction included the following: title, first author, publication year, year of the study, design of the study, population-based study, settings (urban, rural), sample size, data collection procedure, age group of study participants, study places, sampling methods, method of diagnosis used for UDM, and crude prevalence of UDM.

### 2.4. Operational Definition of UDM

Participants do not report a previous diabetes diagnosis, but they found to have diabetes upon tests of their blood glucose and were classified as having UDM or newly diagnosed diabetes. UDM was defined according to the 1999 WHO diagnostic criteria based on fasting blood glucose (FPG) of ≥126 mg/dl or ≥7.0 mmol/l, HbA1c > 6.5%, and OGTT value of ≥11.1 mmol/l 2 h postoral glucose load [31].

### 2.5. Quality Assessment Systems

Evaluation of internal validity of study results, proper sampling methods, clear data collection methods and procedures, reported quality assurance methods (training of data collectors, pretesting, and supervision), and representative sample size were considered study quality indicators.

All quality assessments were entered into standardized data extraction forms. Two authors separately assessed the quality of the studies included using the NIH Quality Assessment Tool for Observational Cohort and Cross-Sectional Studies. Studies that scored a moderate-to-high quality were involved in the analysis. Disagreements of their assessment results were resolved by taking the mean score of the two researchers.

### 2.6. Statistical Data Analysis

The data entry and analysis were done using Excel 2016 and Stata version 11.0 (Stata Corporation, College Station, Texas) software, respectively. The original articles were described using a forest plot and table. The random effects model was used to compute the pooled prevalence and subgroup analysis of UDM. The random effects model was applied to explain any heterogeneity inherent in the meta-analysis. The estimated pooled prevalence rate with its 95% confidence interval (CI) was introduced. Subgroup analyses were performed for residency (rural and urban) and diagnostic methods (OGTT, HbA1c, and FPG).

### 2.7. Heterogeneity and Publication Bias

Statistical heterogeneity was evaluated by Cochran's *Q* test, which shows the amount of heterogeneity between studies, and *I*^2^ statistic. The *I*^2^ statistic was used to estimate the variation (heterogeneity) in the prevalence of UDM among the different African countries, their residence place, and diagnostic methods. *I*^2^ represents the percentage of the total variation in estimated effects across studies due to heterogeneity rather than chance differences. The Begg rank correlation and Egger weighted regression method were used to statistically assess publication bias. *P* < 0.05 is considered suggestive of statistically significant publication bias.

## 3. Results

**Figure 1:**
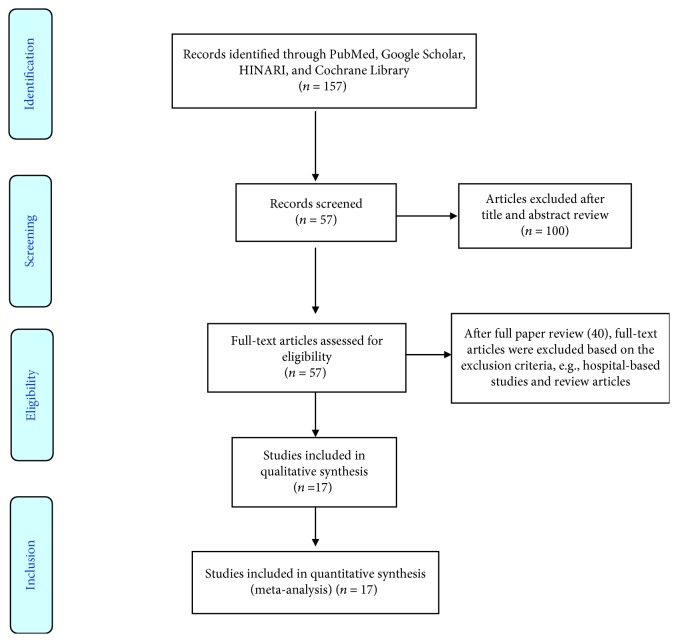
Flow chart for the selection of studies on UDM in Africa.

We identified one hundred fifty-seven articles by the electronic search in MEDLINE/PubMed, Google Scholar, HINARI, and Cochrane Library. Of which, 140 were excluded by the exclusion criteria. Finally, seventeen studies were found to be eligible and included in the meta-analysis (Figure 1).

### 3.1. Study Characteristics

All of the 17 cross-sectional studies included in the meta-analysis were population-based studies. The study populations varied from 382 studies done in Tanzania [16] to 4371 in Ethiopia [21], and these studies were conducted between the years 2011 and 2017. These studies represented the four regions of the continent: West [23–28], East [1, 16–22, 32], Southern [29], and North [30] Africa. The prevalence of UDM varied extensively between studies, ranging from a minimum of 2.3% [20] to a maximum of 18.1% [29], in studies done in South Africa (Table 1). The high prevalence of diabetes (18.1%) in the colored population of South Africa may be due to significant obesity and the economic transition of a community.

### 3.2. Heterogeneity and Publication Bias

The included articles exhibited high heterogeneity according to the *I*^2^ test (*I*^2^ = 93.5%), which is indicative of using a random effects model. In addition, to minimize the random variations between the point estimates of the primary study, subgroup analysis was done based on the residence, region, and method of diagnosis. Moreover, a univariate metaregression model was used by taking the sample size and publication year to identify the possible source of heterogeneity, but none of them was statistically significant. The high heterogeneity may be due to the significant difference in sample size, lifestyle, and genetic factors. The Begg rank correlation (*P* = 0.003) and Egger weighted regression statistics (*P* = 0.004) indicated that there was publication bias. Duval and Tweedie's trim and fill analysis in the random effects model was applied to fix the publication bias.

### 3.3. Outcome Measures

**Figure 2:**
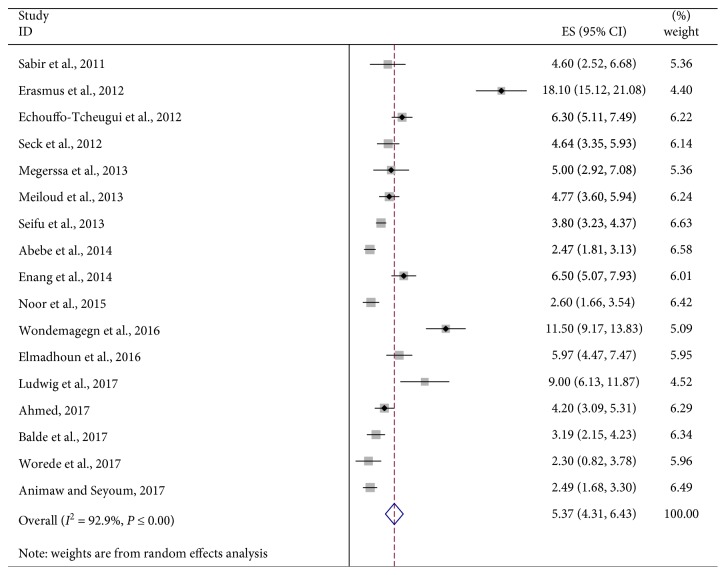
Forest plot of seventeen studies that quantitatively assessed the prevalence of UDM in the African population.

**Figure 3:**
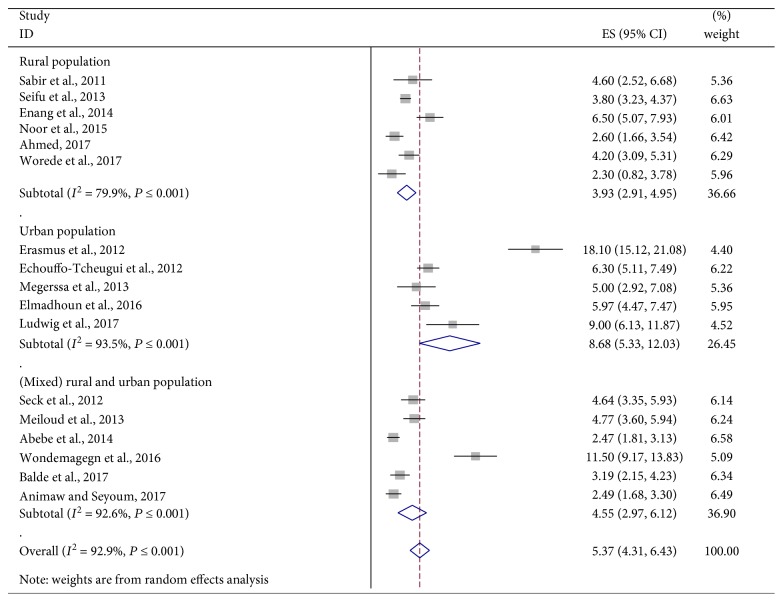
Forest plot of studies that quantitatively assessed the prevalence of UDM in the African population by the residence.

**Figure 4:**
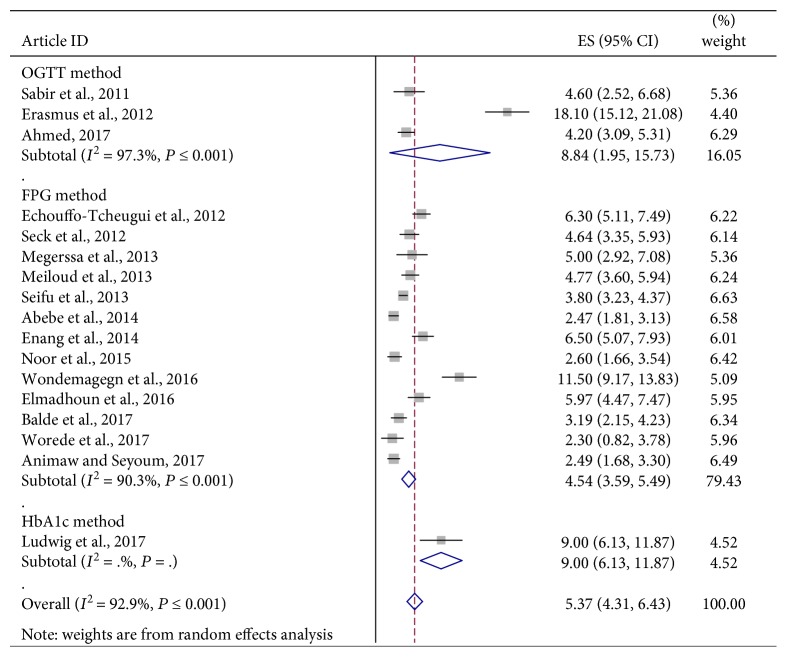
Forest plot of studies that quantitatively assessed the prevalence of UDM in the African population by the diagnostic methods.

The analysis of seventeen studies, according to the DerSimonian-Laird random effects model, revealed that the pooled prevalence of UDM among the African communities was 5.37% (95% CI: 4.31, 6.43) (Figure 2). Subgroup analyses showed that the prevalence of UDM in the urban population (8.68%, 95% CI: 5.33, 12.03) was twice higher than that in the rural population (3.93%, 95% CI: 2.91, 4.95) (Figure 3). In addition, results from subgroup analyses showed that the prevalence of UDM by the OGTT diagnostic method (8.84%, 95% CI: 1.95, 15.73) was higher than that by the FPG diagnostic method (4.54%, 95% CI: 3.59, 5.49) in the study (Figure 4).

## 4. Discussion

The pooled prevalence of UDM among the African communities was 5.3% (95% CI: 4.31, 6.43). The finding from this pooled result indicates that the proportion of UDM cases was inconsistent with the African estimated prevalence of diabetes of 5.7% (19.8 million) in the age group of 20-79 years [33]. This may be due to poor health systems and lack of awareness in the general population. In addition, the pooled prevalence of UDM in Africa was in line with the population-based study in Qatar (5.9%) [11]. This may be because Qatar has a high national prevalence of DM that is due to genetic factors. The African pooled prevalence of UDM was lower than that of studies done in Germany (9.7%) [34], in India (7.2% and 7%) [10, 35], and in Iraq (11%) [36]. The difference might be due to the fact that these studies use OGTT for the definition of DM, while most of the studies included in our meta-analysis were done on FPG, which may underestimate the prevalence of UDM in our study.

However, the prevalence of UDM in this study was higher than that in studies done in Russia (2.9%) [13], China (4.1%) [14], Iran (2.7%) [37], and the USA (0.56%, 4.1%) [5, 38]. This could be due to limited knowledge, attitude, and practice among communities and policy makers in Africa [7, 39].

Based on the subgroup analysis, the prevalence of UDM in the urban population was 8.68% (95% CI: 5.33, 12.03), which was higher than that in studies done in the urban population of India (2.87%) [40], Qatar (5.9%) [11], Indonesia (3.5%) [41], and Iran (5.1%) [12]. The prevalence of UDM in the rural population of Africa was 3.93% (95% CI: 2.91, 4.95) which is higher than that in studies done in the rural population of India (2.87%) [40]. This may be due to lack of awareness towards diabetes diagnosis and symptoms among rural African populations.

In our study, the prevalence of UDM in the urban population was two times higher than that in the rural population, which is different from studies done in Russia that showed that UDM was higher in the rural population than in the urban population (3.8% rural and 2.7% urban) [13]. Similarly, studies have reported a two- to five-fold increase in the risk of diabetes with urban residence [42, 43]. Urbanization is also associated with decreased physical activity energy expenditure, an independent risk factor for the metabolic syndrome [44].

In this study, the prevalence of UDM by the OGTT diagnostic method was higher than that by the FPG diagnostic method. This finding was inconsistent with the recommended diagnostic methods by the American Diabetes Association, which mainly promotes the use of the OGTT for screening of new cases of diabetes [45]. This study highlights the need to develop DM awareness and screening strategies or policy to control DM burden and their complications in African population.

## 5. Conclusion

The results of this regional study confirm the alarmingly high proportions of UDM in many areas of the African population. UDM is harmful and costly, both financially and in terms of complications for individuals and communities and for the health systems. As most of the burden of diabetes was related to its complications, a prevention program based on family history and other targeted screening methods could be an effective way in managing diabetes in African countries.

## 6. Strength and Limitation

This study is the first review and meta-analysis to use a quantitative approach to pool the prevalence of undiagnosed diabetes mellitus in the general population of Africa.

The first limitation of this study was that only English research articles were considered in conducting this region-based review. In addition, all of the studies included in this review were cross-sectional in nature; as a result, the outcome variable might be affected by other confounding variables. Furthermore, in this meta-analysis, most of the studies were from the eastern and western part of Africa. Therefore, some regions may be underrepresented due to the limited number of studies included.

## Figures and Tables

**Table 1:** The characteristics of studies included in the meta-analysis of the prevalence of UDM in African population (*n* = 17).

Article ID	Residence	Country	Region in Africa	Dx lab method	Study design	Sample size (*n*)	Prevalence of UDM (%)
Sabir et al. [27]	Rural	Nigeria	West	OGTT	Cross-sectional	389	4.6
Erasmus et al. [29]	Urban	South Africa	South	OGTT	Cross-sectional	642	18.1
Echouffo-Tcheugui et al. [24]	Urban	Cameroon	Central	FPG	Cross-sectional	1591	6.3
Seck et al. [26]	Rural and urban	Senegal	West	FPG	Cross-sectional	1026	4.64
Megerssa et al. [18]	Urban	Ethiopia	East	FPG	Cross-sectional	422	5
Meiloud et al. [25]	Rural and urban	Mauritania	Western	FPG	Cross-sectional	1278	4.77
Seifu et al. [21]	Rural	Ethiopia	East	FPG	Cross-sectional	4371	3.8
Abebe et al. [17]	Rural and urban	Ethiopia	East	FPG	Cross-sectional	2141	2.47
Enang et al. [28]	Rural	Nigeria	West	FPG	Cross-sectional	1134	6.5
Noor et al. [22]	Rural	Sudan	East	FPG	Cross-sectional	1111	2.6
Wondemagegn et al. [19]	Rural and urban	Ethiopia	East	FPG	Cross-sectional	722	11.5
Elmadhoun et al. [32]	Urban	Sudan	East	FPG	Cross-sectional	954	5.97
Ludwig et al. [16]	Urban	Tanzania	East	HbA1c	Cross-sectional	382	9
Ahmed, 2017	Rural	Egypt	North	OGTT	Cross-sectional	1255	4.2
Balde et al. [23]	Rural and urban	Guinea	West	FPG	Cross-sectional	1100	3.19
Worede et al. [20]	Rural	Ethiopia	East	FPG	Cross-sectional	392	2.3
Animaw and Seyoum [1]	Rural and urban	Ethiopia	East	FPG	Cross-sectional	1405	2.49

## Abbreviations

DM: Diabetes mellitus
UDM: Undiagnosed diabetes mellitus
OGTT: Oral glucose tolerance test
FPG: Fasting blood glucose
CI: Confidence interval
WHO: World Health Organization.
